# The effects of socioeconomic deprivation on colorectal cancer outcomes; a retrospective regional cohort study

**DOI:** 10.1007/s00384-026-05183-x

**Published:** 2026-07-03

**Authors:** Carolyn Cullinane, Roisin Coyne, Emma McNamara, Aaron O. Mahony, Grzegorz Korpanty, Mazen El Bassiouni, Eoghan Condon, Colin Peirce, Patrick O’Donnell, Calvin Coffey, Christina Fleming

**Affiliations:** 1 Department of General and Colorectal Surgery, University Hospital Limerick, UHL Hospital Group, Limerick, Ireland; 2 Department of Medical Oncology, University Hospital Limerick, UHL Hospital Group, Limerick, Ireland; 3 Department of Radiation Oncology, Mater Private Network’s Mid-Western Radiation Oncology Centre, Limerick, Ireland; 4 School of Medicine, University of Limerick, Limerick, Ireland; 5 Health Research Institute, University of Limerick, Limerick, Ireland; 6 Department of Surgical Affairs, Royal College of Surgeons Ireland, Dublin, Ireland

**Keywords:** Colorectal cancer, Social deprivation, Disadvantage

## Abstract

**Background:**

Although colorectal cancer (CRC) survival and outcomes are improving worldwide, there is emerging recognition of the impact of social deprivation on disparities in CRC outcomes. The reasons for the disparities in incidence and mortality amongst different socioeconomic groups are complex and not fully understood. The aim of this study was to explore the impact of socioeconomic deprivation on CRC presentation, treatment course and outcomes.

**Methods:**

A retrospective cohort study of patients with CRC at University Hospital Limerick was performed. A mixed-effects logistic regression analysis approach was utilised to determine the association between socioeconomic deprivation and various CRC outcomes. Data was analysed using IBM SPSS V29.0.2.0 (20).

**Results:**

Six hundred thirty-one patients were eligible for inclusion. Twenty were more likely to present with metastatic disease (OR 1.87, 95% CI 1.002–3.497, *p* = 0.049) or present as an emergency (OR 3.47, CI 2.283–5.274) than non-socioeconomically deprived groups. Of the 466 patients that were treated with curative intent, persons with a lower social deprivation score were more likely to require a stoma (OR 0.25, CI 0.152–0.439) and develop disease recurrence (OR 2.60, CI 1.491–4.467).

**Conclusion:**

Those living in more deprived areas face barriers to accessing timely healthcare and are more likely to present as an emergency or with metastatic CRC. These findings could help inform more effective targeting of public health interventions for CRC.

## Introduction

Colorectal cancer (CRC) is the second most common cause of cancer-related death for men and women in Ireland [1]. In line with other high-income countries, CRC remission rates are considerably higher in Ireland than other regions in the world [2]. Even amongst high-income countries, significant variation in CRC survival outcomes exists possibly due to differences in uptake and coverage of new treatment advances including surgical techniques [3], adjuvant chemotherapy [4], neoadjuvant radiotherapy [5] and multimodal treatment approaches for resectable metastases [6].

Emerging inequalities in cancer care and outcomes are a global concern with disparities existing within as well as between countries. An evolving social gradient for cancer outcomes is becoming apparent with more disadvantaged communities having higher incidence of cancer, more advanced cancer stage and increased mortality for several types of cancer [7, 8]. Previous studies investigating the effect of socioeconomic deprivation on mode and stage of presentation of CRC reported a significant association between social deprivation and advanced CRC stage at presentation [9, 10]. Furthermore, data pertaining to inequalities amongst socioeconomic groups showed that the most socially disadvantaged population groups have a significantly worse prognosis after CRC diagnosis [8]. The reasons for social disparities in CRC incidence and mortality in a universal healthcare system are complex and not completely understood [11–13]. Certain surrogate markers have been reported as access to healthcare, overall health literacy, lack of targeted public health initiatives, limited representation on patient and public forums and a potential complex interaction between different socioeconomic factors [14]. Inequalities in uptake of bowel cancer screening by deprivation and ethnicity have been observed in a UK data set covering 86,850 eligible adults [15]. Ireland’s BowelScreen program offers two yearly faecal immunochemical tests (FIT) to individuals aged 59–70 with a national screening uptake rate of only 46.4% [15]. Efforts to help focus community engagement work and inform research aimed at reducing inequalities are necessary.

Socioeconomic deprivation scores are tools used in medical and public health research to quantify the level of social disadvantage or deprivation experienced by individuals or populations [16]. These scores are often used to study health inequalities and to adjust for socioeconomic confounding in analyses. Different countries use different indices, but most are composite measures that include factors such as income, employment, education, housing and access to services [5]. The Pobal HP Deprivation Index is Ireland’s primary social gradient tool and is based on ten measures including educational attainment, employment status and the numbers living in individual households(Pobal HP Deprivation Index Launched—Pobal).

In Ireland, with a population of approximately five million, there are also disparities in the treatments offered to patients with CRC with a significantly lower proportion of patients surgically treated in more deprived populations [17]. Furthermore, CRC incidence appears to be higher in urban areas and it appears younger populations in such areas are increasingly presenting with more advanced-stage disease [17]. Whilst registry-based databases provide elementary data, the complexities of influencing extrinsic factors are challenging to capture using a cancer register and require further exploration. Therefore, we sought to investigate the real-world impact of social deprivation on CRC outcomes in HSE Midwest.

## Methods

HSE Midwest is a regional health area in the Mid-West of Ireland that delivers acute and elective surgical care for approximately 500,000 people. CRC care across the continuum from diagnosis to surveillance is delivered across four hospitals in the region (three model 2 hospitals, one model 4 hospital). University Hospital Limerick (UHL) is the designated Irish National Cancer Control Programme (NCCP) regional cancer centre.

### Study design

A retrospective cohort study was performed on the records of CRC patients presenting to University Hospital Limerick (UHL). The primary aim of this study was to determine if social deprivation was a poor prognostic indicator for survival in CRC in HSE Midwest independent of tumour stage and co-morbidities. The secondary aims focussed on potential associations between social deprivation and colorectal cancer stage of presentation, treatment strategies and complications.

Ethical approval was obtained from the University of Limerick Hospital group clinical ethics committee (REC Ref:0179). Clinicopathological data, medical co-morbidities, tumour characteristics and Pobal HP Deprivation Index scores were extracted from clinical records into a password-protected hospital database. Participant data was anonymised and handled in line with European data management regulations.

### Patient identification

Patients presenting from January 2021 to November 2024 who were discussed at the regional CRC multidisciplinary meeting (MDM) were included. Patients with histologically confirmed CRC and radiological staging data available were included. Patients were excluded if they were diagnosed with squamous cell carcinoma, neuroendocrine tumour, appendiceal mucinous neoplasms and polyps with high-grade dysplasia or if their HP deprivation score could not be allocated as their address was outside Ireland. Clinical correspondence, radiological investigations and laboratory results relating to each included patient were reviewed to collect clinicopathological features and relevant data.

### Social deprivation index calculation

Socioeconomic disadvantage (SED) scores were determined by entering the patients’ Eircode (a unique code assigned to every address in Ireland) into the publicly available Pobal HP Deprivation Index mapping software. The Pobal HP Deprivation Index is the main index used in Ireland and applied by several government departments, state and semi-state agencies, and voluntary and non-governmental organisations [18]. The Pobal HP Deprivation Index uses data from the Irish national Census of 2022, analysing ten measures of an area’s levels of disadvantage. These include educational attainment, employment status and the numbers of people living in individual households [18].

### Endpoints

The primary study end-point was 5-year disease-free survival (DFS), defined as no radiological or histological evidence of colorectal disease at time of follow-up. Secondary end-points included stage of presentation, stoma formation and emergent presentation defined as bowel obstruction, perforation or acute admission to hospital with a new primary CRC diagnosis. Clinicopathological variables and surrogate markers for social deprivation including age, gender, international protection application, spoken language, ethnicity, registration with a general practitioner and the presence of co-morbidities (i.e. Charleston co-morbidity > 2) were also reported. The growing consensus is that age 50 years is an acceptable arbitrary cutoff to differentiate early-onset CRC from late-onset CRC; therefore, patients were categorised into < 50 years and >/= 50 years for data analysis[19, 20]. Post-operative morbidity was considered a complication classified as Clavien Dindo grade >/= II within 30 days from time of surgery. The number of colorectal cancer diagnoses made via the National Bowel Screening Program was also collated.

### Statistical analysis

For the purpose of this research, patients with a Pobal HP score indicating disadvantage, very disadvantaged or extreme disadvantage were considered to be living in social disadvantage.

Univariable and multivariable logistic regression analyses were used to investigate whether social deprivation was associated with CRC surgical presentation and outcomes. Each risk factor was manually entered into the model, starting with the HP deprivation score and adding each factor in turn. By observing the odds ratios, the 95% confidence interval (CI) for each new factor and the change in the log likelihood statistic, the authors ascertained the independence of each factor. Patient and tumour characteristics were included in multivariable models when associated (*p* < 0.10) in univariable analysis with the surgical outcome of interest.

To analyse the association between social deprivation and 5-year disease-free survival (DFS), univariable and multivariable binomial logistic regression analyses was performed. Multivariable models were adjusted for variables that were associated (*p* < 0.10) in univariable analysis with DFS, age, co-morbidities, nodal involvement, mode of presentation and surgical approach. Single imputation of missing values was performed (< 2% of data were missing) under the missing at random assumption.. Data was analysed using Microsoft Excel and IBM SPSS Statistics ver. 22.0 (IBM Co., Armonk, NY, USA). Two-sided *p* < 0.050 was considered statistically significant. Odds ratios (OR) are presented with 95% confidence intervals (CI).

## Results

### Patient characteristics

Of the total 701 cases reviewed, 631 patients were eligible for inclusion. Patient characteristics are outlined in Table 1. The mean age was 70.9 years (SD 12.41). There was no association between patient age and social deprivation (*p* = 0.075). There were two patients not registered with a general practitioner on the hospital system (0.3%) and seven patients were documented as international protection applicants (1.1%). Over 93% of the patients were Irish (*N* = 589) and English was the first language of 97% of the total study population (*N* = 616). There were 38 patients diagnosed through the national bowel screening programme (6%). Of the total patient population of 631, there were 466 (74%) patients treated with curative intent whilst 165 (26%) were treated with palliative intent after MDT discussion. Two-thirds of the patients had a primary colonic tumour, whilst 30% (*N* = 189) had a rectal tumour and 21 patients (4%) had synchronous tumours. There were 133 patients (21%) who presented emergently and almost half of the patients had significant co-morbidities (42%, *N* = 263).

**Table 1:** Characteristics of included patients

Patient clinical and tumour characteristics	*N* = 631
Age	Mean 70.9 (SD 12.41)
Male	378 (60%)
Female	253 (40%)
Goals of treatment	*N* (%)
Treated with palliative intent	*165 (26%)*
Treated with curative intent	*466 (74%)*
Location	
Rectal	189 (30%)
Colon	421 (66%)
Synchronous	21 (4%)
Emergency presentation	
Yes	133 (21%)
No	498 (79%)
Co-morbidities	
Charlson co-morbidity index = 3/> 3	263 (42%)
Charlson co-morbidity index < 3	368 (58%)
Social Deprivation Score	
Disadvantaged	148 (24%)
*Disadvantaged*	*89*
*Very disadvantaged*	*41*
*Extremely disadvantaged*	*18*
Non-disadvantaged	483 (76%)
Patients treated with curative intent *N* = 466	
Lymph node status	
Positive	172 (36%)
Negative	268(58%)
N/A	26 (6%)
LVI present	121 (26%)
LVI not present	332 (71.2%)
N/A	13 (2.8%)
Stoma	
Yes	183 (39.2%)
No	270 (58%)
N/A	13 (2.8%)
Complication	
Yes	118 (25%)
No	322 (69%)
N/A	26 (6%)
Recurrence	
Yes	69 (15%)
No	394 (84.5%)
Unknown	3 (0.5%)
Follow-up in months Mean	21.17 (SD 13.443)

Of the total patient population, 24% (*N* = 148) were living in disadvantaged (*N* = 89), very disadvantaged (*N* = 41) or extremely disadvantaged (*N* = 18) areas according to their Pobal HP Deprivation Index. Amongst the patients treated with curative intent, lymph node positivity was present in 172 patients (36%) and over one quarter of patients had lymphovascular invasion (LVI) evident in their specimens (*n* = 121). A minimally invasive surgical approach was utilised in 346 patients (74%), 10 patients entered the rectal watch and wait program (2.1%) and 183 (39%) had surgery with a resultant stoma. The mean follow-up time was 21 months and in that time 15% (*N* = 69) of patients had histologically or radiologically confirmed disease recurrence. The clinicopathological characteristics of patients living in social deprivation are presented in Table 2.

**Table 2:** Clinicopathological characteristic on patients living in social disadvantage

Total population cohort *N* = 631			
	Living in social disadvantage (*N*, %) (148)	Not living in social disadvantage (*N*, %) (483)	*p*-value
Age < 50 years (*N* = 44)	12 (8%)	32 (6.6%)	0.663
Age > 50 years (*N* = 587)	136 (92%)	451 (93.4%)	
Emergency presentation (*N* = 133)	58 (39.2%)	75 (15.5%)	< 0.001
Non-emergent presentation (*N* = 498)	90 (60.8%)	408 (84.5%)	
Metastatic disease (*N* = 138)	46 (31%)	92 (19%)	0.003
Non-metastatic disease (*N* = 493)	102 (69%)	391 (81%)	
Charlson co-morbidity index > 2 (*N* = 263)	78 (52.7%)	185 (38.3%)	0.003
Charlson co-morbidity index 2/< 2(*N* = 368)	70 (47.3%)	298 (61.7%)	
Synchronous (*N* = 21)	5 (3.4%)	16 (3.3%)	1.000
Non-synchronous (*N* = 610)	143 (96.6%)	467 (96.7%)	
Rectal (including those with synchronous) (*N* = 210)	53 (35.8%)	157 (32.5%)	0.518
(*N* = 421)	95 (64.2%)	326 (67.5%)	
Population treated with curative intent *N* = 466			
	Living in social disadvantage (*N*, %) (102)	Not living in social disadvantage (*N*, %) (364)	
Node-positive disease (*N* = 172)	42 (41%)	130 (36%)	0.454
Node-negative disease (*N* = 268)	56 (55%)	212 (58%)	
Unknown (*N* = 26) (W&W, awaiting, endoscopic)	4 (4%)	22 (6%)	
LVI present (*N* = 121)	36 (35.3%)	85 (23.4%)	0.030
No LVI present (*N* = 332)	65 (63.7%)	267 (73.4%)	
Unknown (*N* = 13) (W&W, awaiting)	1 (1%)	12 (3.2%)	
Stoma *N* = 183	64 (62.7%)	119 (32.7%)	< 0.001
No stoma *N* = 270	36 (35.3%)	234 (64.3%)	
NA (W&W, awaiting) (*N* = 13)	2 (2%)	11 (3%)	
Minimally invasive surgery (*N* = 346)	67 (65.7%)	279 (76.6%)	0.018
Open surgery (*N* = 107)	33 (32.3%)	74 (20.3%)	
Watch and wait (*N* = 10)	1 (1%)	9 (2.5%)	
Awaiting resection (*N* = 3)	1 (1%)	2 (0.6%)	
Post-op morbidity (*N* = 118)	35 (34.3%)	83 (22.8%)	0.018
No post-op morbidity (*N* = 322)	60 (58.8%)	262 (72%)	
NA (W&W, awaiting, endoscopic) (*N* = 26)	7 (6.9%)	19 (5.2%)	
Recurrence (*N* = 69)	29 (28.4%)	40 (11%)	< 0.001
No recurrence (*N* = 394)	72 (70.6%)	322 (88.4%)	
NA (*N* = 3)	1 (1%)	2 (0.6%)	

LVI*, lymphovascular invasion

Watch and wait (W&W)* *N* = 10, awaiting colorectal resection** *N* = 3, and endoscopic/transanal excision*** *N* = 13. *p*-values were calculated using *χ*^2^ tests comparing living in social disadvantage vs not living in social disadvantage

### Social deprivation and presentation of CRC

Of the total population of 631 patients, 133 (21%) patients presented emergently. Patients who lived in disadvantaged, very disadvantaged or extremely disadvantaged areas (*N* = 148, 24%) were more likely to present as an emergency (OR 3.85, 95% CI 2.235–6.643, *p* < 0.001). Similarly, patients who lived in more extreme areas of disadvantage (very/extremely disadvantaged) had a significantly increased odds of presenting as an emergency compared to persons from all other areas (OR 7.95, 95% CI 3.94–16.04, *p* < 0.001). Other clinicopathological variables independently associated with a colorectal emergency presentation were Charlson co-morbidity index > 2 (CCI > 2) (OR 1.78, 95% CI 1.029–3.092, *p* = 0.039) and age younger than 50 years old at diagnosis (OR 3.60, 95% CI 1.565–7.624, *p* = 0.002). Those presenting as an emergency were also more likely to have node-positive CRC (OR 1.74, 95% CI 1.229–2.456, *p* = 0.002) (Table 3).

**Table 3:** Multivariate analysis associated with emergency presentation and metastatic presentation

	Emergency presentation Univariate	Emergency presentation Multivariate	Metastatic disease Univariate	Metastatic disease Multivariate
Disadvantaged	OR 3.72, 95% CI 2.464–5.628, ***p*** < 0.001	OR 3.85, 95% CI 2.235–6.643, ***p*** < 0.001	OR 2.49, 95% CI 1.647–3.783, ***p*** < 0.001	OR 1.87, 95% CI 1.002–3.497, ***p*** = 0.049
Very/extremely disadvantaged	OR 7.04, 95% CI 3.61–13.74, ***p*** < 0.001	OR 7.95, 95% CI 3.94–16.04, ***p*** < 0.001	OR 2.14, 95% CI 0.69–6.63, *p* = 0.185	
Charlson co-morbidity index > 2	OR 2.19, 95% CI 1.483–3.230, ***p*** < 0.001	OR 1.78, 95% CI 1.029–3.092, ***p*** = 0.039	OR 2.01, 95% CI 1.370–2.953, ***p*** < 0.001	OR 1.83, 95% CI 1.229–2.714, ***p*** = 0.003
Age (< 50 years)	OR 3.64,95% CI 1.746–7.611, ***p*** < 0.001	OR 3.60, 95% CI 1.565–7.624, ***p*** = 0.002	OR 3.39, 95% CI 2.031–7.844, ***p*** < 0.001	OR 2.58, 95% CI 1.177–5.682, ***p*** = 0.018
Synchronous	OR 1.96, 95% CI 0.773–4.949, *p* = 0.157		OR 0.59, 95% CI 0.170–2.741, *p* = 0.404	
Node-positive disease	OR 1.73, 95% CI 1.246–2.392, ***p*** = 0.001	OR 1.74, 95% CI 1.229–2.456, ***p*** = 0.002	OR 3.33, 95% CI 2.025–5.478, ***p*** < 0.001	OR 3.31, 95% CI 2.007–5.472, ***p*** < 0.001
Rectal cancer	OR 1.25, 95% CI 0.831–1.884, *p* = 0.282		OR 1.14, CI 0.765–1.705, *p* = 0.516	
International protection applicant	OR 1.53, 95% CI 0.294–7.918, *p* = 0.613		OR 0.41, 95% CI 0.48–3.54, *p* = 0.495	

A total of 138 (22%) patients presented with metastases. Living in a disadvantaged area was independently associated with presenting with metastatic disease (OR 1.87, 95% CI 1.002–3.497, *p* = 0.049). Similarly, having a CCI > 2 (OR 1.83, 95% CI 1.229–2.714, *p* = 0.003) and aged younger than 50 at diagnosis (OR 2.58, 95% CI 1.177–5.682, *p* = 0.018) were both associated with metastatic disease at presentation. Presenting emergently was associated with metastatic disease on univariate analysis (OR 1.78, 95% CI 1.161–2.724, *p* = 0.008) but not multivariate analysis (OR 0.92, 95% CI 0.587–1.455, *p* = 0.734) (Table 3).

### Social deprivation and surgical outcomes

There were 466 (74%) patients treated with curative intent after multidisciplinary team discussion, 22% (*N* = 103) of which were living in disadvantaged (*N* = 61), very disadvantaged (*N* = 27) or extremely disadvantaged (*N* = 15) areas. A minimally invasive surgical approach was performed in 346 (74.2%) cases and 107 (23%) patients had an open operation. There were 10 (2.1%) patients enrolled in the rectal cancer watch and wait program following a complete clinical response to neoadjuvant therapy, 13 patients underwent endoscopic or local tumour resection (2.8%) and 3 (0.6%) patients were receiving total neoadjuvant treatment at the time of data collection. Patients from socially deprived areas were more likely to present with a CCI > 2 (OR 1.97, CI 1.26–3.09, *p* = 0.003). Patients who presented emergently had a CCI > 2, had node-positive disease or synchronous tumours and lived in socially disadvantaged areas were all significantly less likely to undergo minimally invasive surgery (Table 4).

**Table 4:** Multivariate analysis associated with minimally invasive surgery, stoma formation and post-op complications

	Minimally invasive surgery Univariate	Minimally invasive surgery Multivariate	Stoma formation Univariate	Stoma formation Multivariate	Post-op complication Univariate	Post-op complication Multivariate
Disadvantaged	OR 0.53, 95% CI 0.326–0.856, ***p*** = 0.010	OR 0.90, 95% CI 0.506–1.617, *p* = 0.735	OR 4.29, 95% CI 2.665–6.896, ***p*** < 0.001	OR 3.48, 95% CI 1.968–6.151, ***p*** < 0.001	OR 1.89, 95% CI 1.161–3.075, ***p*** = 0.010	OR 1.11, 95% CI 0.697–1.759, *p* = 0.66
Very/extremely disadvantaged	OR 0.35, 95% CI 0.127–0.197, ***p*** = 0.047	OR 0.82, 95% CI 0.243–2.739, *p* = 0.741	OR 4.17, 95% CI 1.310–13.290, ***p*** = 0.016	OR 2.51, 95% CI 0.595–10.590, *p* = 0.210	OR 1.09, 95% CI 0.337–3.57, *p* = 0.878	
Charleston Morbidity Index > 2	OR 0.54, 95% CI 0.350–0.841, ***p*** = 0.006	OR 0.60, 95% CI 0.364–0.984, ***p*** = 0.043	OR 2.56, 95% CI 1.713–3.811, ***p*** < 0.001	OR 3.03, 95% CI 1.851–4.964, ***p*** < 0.001	OR 1.73, 95% CI 1.116–2.674, ***p*** = 0.014	OR 1.55, 95% CI 0.981–2.460, *p* = 0.060
Rectal cancer	OR 0.67, 95% CI 0.415–1.018, *p* = 0.102		OR 1.23, 95% CI 0.150–1.358, *p* = 0.78		OR 1.56, 95% CI 0.950–2.552, *p* = 0.079	
Age (< 50 years)	OR 0.89, 95% CI 0.418–1.883, *p* = 0.755		OR 3.65, 95% CI 1.746–7.611, ***p*** < 0.001	OR 3.39, 95% CI 1.290–8.881, ***p*** = 0.013	OR 1.84, 95% CI 0.908–3.732, *p* = 0.091	
Emergency presentation	OR 0.13, 95% CI 0.078–0.228, ***p*** < 0.001	OR 0.15, 95% CI 0.085–0.276, ***p*** < 0.001	OR 6.25, 95% CI 3.483–11.198, ***p*** < 0.001	OR 6.87, 95% CI 3.492–13.512, ***p*** < 0.001	OR 4.51, 95% CI 2.653–7.671, ***p*** < 0.001	OR 4.08, 95% CI 2.347–7.088, ***p*** < 0.001
Synchronous tumour	OR 0.33, 95% CI 0.124–0.879, ***p*** = 0.027	OR 0.37, 95% CI 0.123–1.102, *p* = 0.074	OR 3.64, 95% CI 1.261–10.521, ***p*** = 0.017	OR 3.52, 95% CI 0.855–14.491, *p* = 0.81	OR 3.25, 95% CI 1.222–8.634, ***p*** = 0.018	OR 2.90, 95% CI 0.860–9.972, *p* = 0.086
Node-positive disease	OR 0.58, 95% CI 0.425–0.783, ***p*** < 0.001	OR 0.66, 95% CI 0.470–0.922, ***p*** = 0.015	OR 0.90, 95% CI 0.680–1.192, *p* = 0.46		OR 0.96, 95% CI 0.700–1.306, *p* = 0.779	

Of the 466 patients treated with curative intent, 39.3% (*n* = 183) had stoma-forming surgery. Patients living in a socially disadvantaged area were over three times more likely to require a stoma (OR 3.48, 95% CI 1.968–6.151, *p* < 0.001). Other clinicopathological factors independently associated with stoma requirement included CCI > 2 (OR 3.03, 95% CI 1.851–4.964, *p* < 0.001), age less than 50 at diagnosis (OR 3.39, 95% CI 1.290–8.881, *p* = 0.013) and an emergent presentation (OR 6.87, 95% CI 3.492–13.512, *p* < 0.001). The presence of synchronous disease was also associated with stoma formation on univariate analysis (OR 3.64, 95% CI 1.261–10.521, *p* = 0.017) but not multivariate analysis (OR 3.52, 95% CI 0.855–14.491, *p* = 0.81) (Table 4).

The overall post-operative complication rate (Clavien Dindo grade >/= II) was 25%. Social deprivation, CCI > 2, an emergent presentation and synchronous tumours were all associated with post-op complications; however, only an emergent presentation (OR 4.08, 95% CI 2.347–7.088, *p* < 0.001) was independently associated with an increased risk of post-op complications on multivariate analysis (Table 4).

### Social deprivation and colorectal cancer outcomes

In the mean follow-up period of 21 months, 15% (*N* = 69) of patients had histologically or radiologically confirmed CRC recurrence. SD (OR 2.67 95% CI 1.534–4.630, *p* < 0.001), age less than 50 at diagnosis (OR 2.64, 95% CI 1.238–5.641, *p* = 0.012), emergency presentation (OR 5.39, 95% CI 3.022–9.567, *p* < 0.001), nodal positivity (OR 3.80, 95% CI 2.614–5.507, *p* > 0.001) and LVI (OR 3.87, 95% CI 2.253–6.634, *p* < 0.001) were all associated with disease recurrence on univariate analysis. However, only emergent presentation (OR 2.49, 95% CI 1.198–5.184, *p* = 0.015) and nodal positivity (OR 3.01, 95% CI 1.968–4.598, *p* < 0.001) remained significant on multivariate analysis. Furthermore, minimally invasive surgery was associated with reduced risk of disease recurrence on multivariate analysis (OR 0.48, 95% CI 0.252–0.930, *p* = 0.030) (Table 5).

**Table 5:** Multivariate analysis associated with disease recurrence

	Univariate	Multivariate
Disadvantaged	OR 2.67, 95% CI 1.534–4.630, ***p*** < 0.001	OR 1.62, 95% CI 0.826–3.179, *p* = 0.160
Very/extremely disadvantaged	OR 2.95, 95% CI 0.977–8.925, ***p*** = 0.05	OR 1.97, 95% CI 0.565–6.880, *p* = 0.287
Age (< 50 years)	OR 2.64, 95% CI 1.238–5.641, ***p*** = 0.012	OR 2.27, 95% CI 0.912–5.670, *p* = 0.078
Emergency	OR 5.39, 95% CI 3.022–9.567, ***p*** < 0.001	OR 2.49, 95% CI 1.198–5.184, ***p*** = 0.015
Charlson co-morbidity index > 2	OR 1.31, 95% CI 0.769–2.221, *p* = 0.323	
Synchronous	OR 1.21, 95% CI 0.339–4.340, *p* = 0.766	
Rectal	OR 1.84, 95% CI 0.989–3.408, *p* = 0.054	OR 1.40, 95% CI 0.652- 2.99, *p* = 0.390
Nodal positivity	OR 3.80, 95% CI 2.614–5.507, ***p*** > 0.001	OR 3.01,95% CI 1.968–4.598, ***p*** < 0.001
LVI	OR 3.87, 95% CI 2.253–6.634, ***p*** < 0.001	OR 1.60, 95% CI 0.840–3.063, *p* = 0.153
Min-invasive surgery	OR 0.29, 95% CI 0.162–0.475, ***p*** < 0.001	OR 0.48, 95% CI 0.252–0.930, ***p*** = 0.030

## Discussion

The results of this study show that SED is associated with less favourable colorectal cancer outcomes. In all new colorectal cancer cases diagnosed in our institution from January 2021 to November 2024, SED was associated with an increased odds of presenting as an emergency or with metastatic disease. These associations were independent of age, CCI > 2 and tumour characteristics suggesting that social disadvantage–specific differences in stage of presentation cannot be explained by other social demographic variables. In terms of the impact of SED on surgical outcomes, patients not categorised as socially deprived were less likely to have a stoma, undergo open surgery or develop post-operative complications. In addition, there was a trend towards higher incidences of colorectal cancer recurrence in patients categorised as socially deprived; however, this did not reach statistical significance in multivariate analysis.

The negative impact of SED on CRC outcomes is well-documented with people from lower socioeconomic backgrounds having poorer outcomes throughout the entire CRC care continuum [21, 22]. CRC disproportionately affects people from lower socioeconomic backgrounds and some racial minorities potentially due to social risk factors (smoking, sedentary lifestyle) and limited access to risk-reducing interventions such as chemo-prevention and changes to the microbiome [23, 24].

Furthermore, our study also found a significant association between SED and advanced stage of CRC presentation. An explanation for the higher incidence of more advanced CRC stage and emergency presentations according to social gradient centres on early detection interventions such as screening and an individual’s willingness to engage with healthcare services [21]. Screening is a well-established intervention for reducing CRC death [25]. Evidence suggests that people from lower-education backgrounds are less likely to undergo screening than people from higher-education backgrounds [26]. Previous modelling studies report a possible 19% difference in CRC mortality according to screening uptake [27]. Whilst an association between screening uptake and social disadvantage was not observed in this study, this could be due to the small overall numbers of patients who were diagnosed through the National Bowel Screening program (6%, *N* = 38). Appreciably, the numbers are small but highlight the importance of targeted public health initiatives and access to primary care physicians to increase screening uptake.

Previous international studies investigating the relationship between cancer stage at diagnosis and SED failed to reach any conclusion due to inconsistencies in staging information in population-based cancer registries [28, 29]. Similarly, reports from the National Cancer Registry in Ireland reported no association between SED and CRC incidence stage of presentation and the presence of co-morbidities [30]. Whilst these large registry-based studies are informative, they are unable to unravel the influence of specific confounders such as age, the presence of co-morbidities, the location of the primary cancer (rectal or colon) or whether the presentation was emergent. The present study demonstrates that SED is independently associated with advanced stage of CRC presentation and an increased odds of presenting emergently.

Social disparity–specific differences in surgical treatment characteristics were detected in this study. Minimally invasive surgical (MIS) approaches were utilised in 346 patients (74%). Considering the advanced stage of presentation and emergent mode of presentation, it is unsurprising that patients living in deprivation had an increased odds of undergoing open surgery. SED was associated with an elevated risk of developing post-operative complications on univariate analysis but failed to achieve significance on multivariate analysis. The differences in surgical treatment and outcomes observed in the present study are in agreement with findings from previous studies, with patients with CRC and lower socioeconomic status more likely to undergo open surgery[31, 32] and a higher probability of developing a post-operative complication [32]. The disparities in CRC surgical outcomes observed in studies from the USA could be explained by the inclusion of uninsured patients with limited access to high-quality medical care [31]. In Europe, a Dutch study group reported significantly higher CRC laparotomy rates and anastomotic leak rates in patients with lower socioeconomic status [33]. Furthermore, a recent study from Sweden, which has universal healthcare coverage, reported that rectal cancer patients with the highest level of education and the highest household income had an increased probability of receiving minimally invasive surgery (MIS) [34]. Considering the superior short-term post-operative outcomes associated with MIS such as less pain, less need for analgesics, faster recovery and shorter hospital stay, at first glance an open surgery rate of 56.4% appears surprising in such a contemporary data set [34]. However, the authors clarify that adoption of MIS techniques has readily increased over the last decade so that in 2023, 85% of rectal cancer surgeries were performed minimally invasive. Whilst country-specific outcomes could be attributed to variations in healthcare systems, the reported disparities amongst populations in publicly funded health systems are more multifaceted. Ireland’s two-tier health service refers to a system where people can access healthcare through two parallel pathways: the public system and the private system. Both operate largely within the same hospitals but provide different levels of access and patient choice. Historically, privately insured patients were offered consultant-led care which could account for the social disparity in surgical technique and outcomes evident in this study. It is likely that social disparities in healthcare are more complex than access to services alone with health-seeking behaviours, health-related lifestyle behaviours, socioeconomic position and health literacy all intrinsically involved [28]. Emerging data from the USA suggests that 30% of adults have low health literacy, defined as the degree to which individuals are able to find, understand and use information and services to inform health-related decisions and actions for themselves and others [35]. Encouraging healthcare systems to equitably enable patients to find, understand and use information and services to inform health-related decision and training staff to confirm comprehension in a non-judgemental way could potentially bridge the equity gap in publicly funded systems [35].

To the best of our knowledge this is the first study to report an association between SEDe and stoma formation. Patients living in SED were three times more likely (OR 3.48, 95% CI 1.968–6.151, p < 0.001) to require a stoma than non-socially deprived cohorts. Stoma-forming surgery is reported to change the way people relate to their social environment and connect with others, creating self-consciousness and impeding social confidence and autonomy[36]. Whilst stoma formation is required more frequently in advanced disease and more co-morbid patients, understanding the social implications of stoma-forming surgery amongst more marginalised groups could help clinicians to provide responsive and appropriate support to facilitate social rehabilitation[36].

The negative impact of SED on CRC survival outcomes is well established in the literature [37–39]. The main factors associated with worse survival outcomes in patients with lower SED have been suggested to be the higher number of co-morbidities, more unfavourable health-related behaviours, more advanced stage at presentation and access inequalities to treatment [37, 40, 41]. In the present study, SED was associated with poorer disease-free survival on univariate analysis. In accordance with what has been hypothesised previously, the observed social disparity associated with poorer disease-free survival outcomes appeared dependent on other clinical confounders [42]. When adjusted for age, social disadvantage, co-morbidities and tumour-specific factors, only node positivity, open surgery and an emergency presentation were significantly associated with poorer DFS. In line with international data, the proportion of CRC presenting emergently in this study was 21% [43]. Whilst factors including more advanced disease stage and higher American Society of Anaesthesiology (ASA) grade at presentation may contribute to the association between emergency CRC presentations and worse outcomes, recent research suggests that emergency presentation remains an independent poor prognostic indicator following curative colorectal resection[44]. The significant association between SED and emergency presentation of CRC likely contributes to the poorer DFS outcomes in this setting. Furthermore, it is likely that there is a complex interplay between various patient and tumour variables to explain the socioeconomic gradient evident in CRC survival outcomes in this study.

A strength of this study is that it included an unselected CRC group and despite the retrospective nature of the study, missing data was < 2% and comprehensive data on disease stage, co-morbidities, tumour characteristics, surgical treatments, complications and disease recurrence were available. Also whilst this was a single regional health area study, it was performed in the most socially diverse area in Ireland which also contains the most socially deprived area nationally [45]. A limitation of the study may be the use of an indicator of socioeconomic status such as the Pobal HP social deprivation score based on the postcode (Eircode) of the residential area. The Pobal HP Deprivation Index is Ireland’s primary social gradient tool; however, it does have limitations in that not all surrogates of health inequalities are considered, e.g. literacy, language and access to primary care facilities. Another considerable limitation of this research is that patients with a Pobal HP score indicating disadvantage, very disadvantaged or extreme disadvantage were all considered to be living in social disadvantage for outcome analysis. A subgroup analysis was performed on the very and extremely disadvantaged cohort; however, the small sample size deemed many of the results statistically insignificant. Furthermore, surgical approaches were grouped into minimally invasive and open/conversion to open due to the relatively small study sample. Future studies will sub-categorise minimally invasive techniques into laparoscopic, robotic and transanal to determine whether newer surgical techniques have yielded more favourable survival outcomes as demonstrated by the recently published REAL trial [46].

To conclude, this study demonstrated a significant disparity in CRC stage of presentation, mode of presentation and surgical-specific outcomes due to SED. These differences appeared independent of age, co-morbidities and tumour-specific factors. Findings from this study will inform more effective targeting of public health interventions for CRC and the data will support stakeholders to equitably enable patients living in social deprivation access services available to them and make informed health-related decisions to potentially bridge the health equity gap.

## Data Availability

Data pertaining to this study has been pseudoanonymised to protect the study participants. A request to review study data can be made to the authors.
